# Co-production of hydrogen and ethyl acetate in *Escherichia coli*

**DOI:** 10.1186/s13068-021-02036-3

**Published:** 2021-10-01

**Authors:** Anna C. Bohnenkamp, René H. Wijffels, Servé W. M. Kengen, Ruud A. Weusthuis

**Affiliations:** 1 Bioprocess Engineering, Wageningen University and Research, Droevendaalsesteeg 1, 6708 PB Wageningen, The Netherlands; 2 Faculty of Biosciences and Aquaculture, Nord University, 8049 Bodø, Norway; 3 Laboratory of Microbiology, Wageningen University and Research, Stippeneng 4, 6708 WE Wageningen, The Netherlands

**Keywords:** Ethyl acetate, Hydrogen, Co-production, Fermentation, *Escherichia coli*, Eat1, Formate hydrogen lyase

## Abstract

**Background:**

Ethyl acetate (C_4_H_8_O_2_) and hydrogen (H_2_) are industrially relevant compounds that preferably are produced via sustainable, non-petrochemical production processes. Both compounds are volatile and can be produced by *Escherichia coli* before. However, relatively low yields for hydrogen are obtained and a mix of by-products renders the sole production of hydrogen by micro-organisms unfeasible. High yields for ethyl acetate have been achieved, but accumulation of formate remained an undesired but inevitable obstacle. Coupling ethyl acetate production to the conversion of formate into H_2_ may offer an interesting solution to both drawbacks. Ethyl acetate production requires equimolar amounts of ethanol and acetyl-CoA, which enables a redox neutral fermentation, without the need for production of by-products, other than hydrogen and CO_2_.

**Results:**

We engineered *Escherichia coli* towards improved conversion of formate into H_2_ and CO_2_ by inactivating the formate hydrogen lyase repressor (*hycA*), both uptake hydrogenases (*hyaAB*, *hybBC*) and/or overexpressing the hydrogen formate lyase activator (*fhlA*), in an acetate kinase (*ackA*) and lactate dehydrogenase (*ldhA*)-deficient background strain. Initially 10 strains, with increasing number of modifications were evaluated in anaerobic serum bottles with respect to growth. Four reference strains *ΔldhAΔackA*, *ΔldhAΔackA p3-fhlA, ΔldhAΔackAΔhycAΔhyaABΔhybBC and ΔldhAΔackAΔhycAΔhyaABΔhybBC p3-fhlA* were further equipped with a plasmid carrying the heterologous ethanol acyltransferase (Eat1) from *Wickerhamomyces anomalus* and analyzed with respect to their ethyl acetate and hydrogen co-production capacity. Anaerobic co-production of hydrogen and ethyl acetate via Eat1 was achieved in 1.5-L pH-controlled bioreactors. The cultivation was performed at 30 °C in modified M9 medium with glucose as the sole carbon source. Anaerobic conditions and gas stripping were established by supplying N_2_ gas.

**Conclusions:**

We showed that the engineered strains co-produced ethyl acetate and hydrogen to yields exceeding 70% of the pathway maximum for ethyl acetate and hydrogen, and propose in situ product removal via gas stripping as efficient technique to isolate the products of interest.

**Supplementary Information:**

The online version contains supplementary material available at 10.1186/s13068-021-02036-3.

## Background

Esters are a diverse group of compounds important not only for the food industry, but also for various industrial purposes [8]. Ethyl acetate is among the most relevant esters with respect to industrial use. It is considered relatively environmentally friendly and thus a popular solvent used in paints and adhesives, and other applications.

Yeasts are natural producers of a variety of esters, including ethyl acetate. Efforts have been made to understand and direct ester production and composition, focusing on bulk producers of ethyl acetate, including *Kluyveromyces marxianus* (*K. marxianus*) and *Wickerhamomyces anomalus* (*W. anomalus*) [7, 15, 26]. Especially *K. marxianus* has been exploited and optimized with respect to efficient ethyl acetate production. In fermentations on whey-based medium a yield of 0.265 *g*_ethyl acetate_/*g*_sugar_, corresponding to 50% of the maximum yield, was reached in a 70-L reactor, demonstrating the scalability of the system [13]. Recently, we have shown that a heterologous expression system in *Escherichia coli* (*E. coli*) can compete with natural producers in terms of ethyl acetate yields [3]. A streamlined *E. coli* strain harboring a truncated ethanol acetyltransferase (*eat1*) gene from *W. anomalus* reached 72% of the maximum pathway yield on glucose under anoxic conditions. This is the highest reported yield to date.

**Fig. 1:**
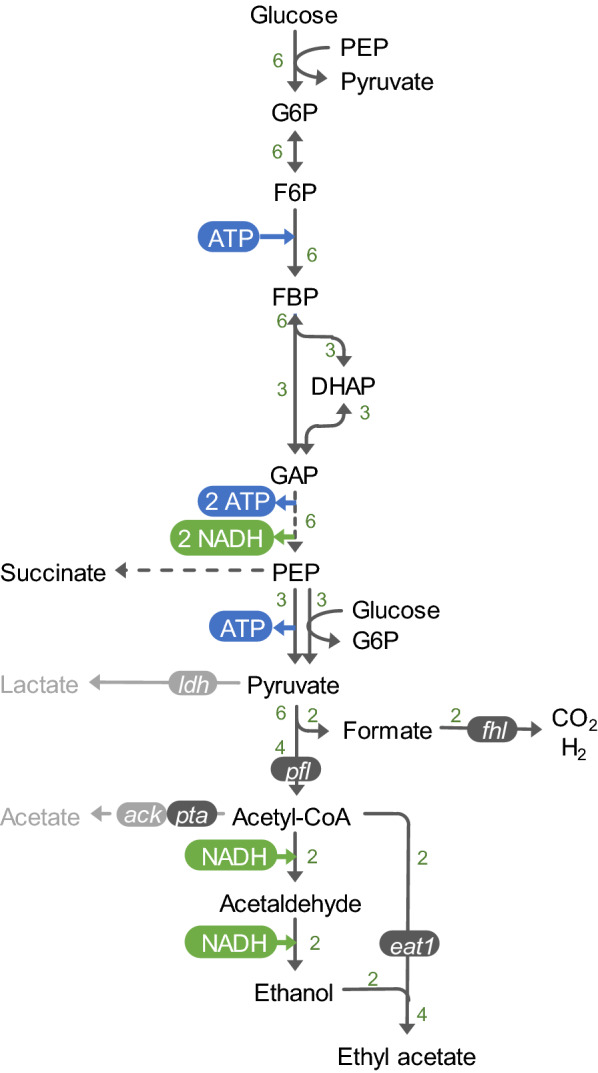
Schematic representation of anaerobic ethyl acetate production from glucose in *E. coli* via the Embden–Meyerhof–Parnas (EMP) pathway with hydrogen co-production. Lactate and acetate formation is limited by ack and ldh inactivation. Heterologous alcohol acetyltransferase Eat1 generates ethyl acetate from ethanol and acetyl-CoA. Hydrogen co-production is achieved via formate hydrogen lyase (Fhl). *Ack* acetate kinase, *DHAP* dihydroxyacetone phosphate, *eat1* ethanol acetyltransferase, *FBR* fructose 1,6-bisphosphate, *F6P* fructose 6-phosphate, *G6P* glucose 6-phosphate, *PEP* phosphoenolpyruvate, *GAP* glyceraldehyde 3-phosphate, *pta* phosphate acetyltransferase

In contrast to yeasts that use pyruvate decarboxylase to convert pyruvate to acetaldehyde, *E. coli* uses pyruvate formate lyase to produce acetyl-CoA during anaerobic conditions [3]. This ultimately results in a redox and carbon balanced pathway under anoxic conditions, contributing to the overall efficiency of the process as less carbon is lost to biomass or respiration [33]. However, as *E. coli* uses pyruvate formate lyase, one mole of formate is coproduced with every conversion of pyruvate into acetyl-CoA, coproducing two moles of formate per generated mole of ethyl acetate (Fig. 1).

Formate is accumulating during the fermentation process, acidifying the medium and causing inhibiting effects on the *E. coli* cells. While the acidification of the medium can be prevented by pH-control of the reactor, buildup of formate to inhibiting concentrations may nevertheless negatively affect performance of the system. Formate concentrations below 100 mM already severely hamper *E. coli* growth, and concentrations of 50 mM have been reported to cause growth inhibition of 50% [37]. One way *E. coli* counteracts these negative side-effects of formate, is by converting it to CO_2_ and H_2_ by a membrane-bound formate hydrogen lyase (Fhl) after formate concentrations exceed a certain threshold [20].

Hydrogen is considered an attractive, environmentally friendly energy carrier, but 95% of the current production is still derived from non-renewable resources [1, 22]. In order to benefit from hydrogen as future fuel also its production needs to rely on sustainable methods paving the path for green or bio-hydrogen [4, 10, 28]. Regarding microbial hydrogen production attention has been paid to increasing yields and productivity by means of genetic engineering, with a strong focus on *E. coli*. While *E. coli* primarily secretes formate and naturally is a poor hydrogen producer, the complexity and transcriptional regulation of the Fhl complex with the involvement of around 15 genes is well understood [2, 25, 39]. Due to its annotated genome and well established genetic engineering tools, several targets and strategies for improving hydrogen production have been identified [18].

Several studies used formate as substrate for the production of bio-hydrogen from *E. coli* [24, 35]. Inactivating the Fhl repressor *hycA* was among the first modifications to promote Fhl activity, thus enhancing hydrogen production [24]. Combining *hycA* deactivation and overexpression of the formate hydrogen lyase transcriptional activator (FhlA) further improved strain performance [35]. In addition, Maeda and colleagues studied the effect of various modifications concerning hydrogen production and uptake, extensively [16]. They found that besides inactivating *hycA* and overexpressing *fhlA*, inactivation of hydrogen uptake by knocking out hydrogenase 1 (*hyaB*) and 2 (*hybC*) further benefitted hydrogen production. Moreover, inactivating *hycA hyaB hybC* together with inactivating the formate transporter *focA* did not impact growth of *E. coli* under aerobic conditions, while leading to an almost fivefold increased hydrogen production capacity with respect to wild-type *E. coli* [17].

However, to date microbial hydrogen production with sole focus on generation of bio-hydrogen is considered rather unfeasible mainly due to the low conversion efficiency and low maximum yields obtained [22]. Therefore, coupling it to the production of another relevant product may improve the overall feasibility of such process as shown with the example of ethanol [12, 28, 29]. However, ethanol and hydrogen are competing for electrons and maximum yields for one product will automatically decrease the achievable yield for the other product.

This study investigates in how far redox-balanced co-production can benefit the bio-based generation of two industrially relevant compounds with respect to yields and rates. Here, we describe the efficient co-production of ethyl acetate and bio-hydrogen using an engineered *E. coli* strain, while restricting product accumulation by in situ product removal.

## Results

### Increasing hydrogen gas production

A series of modifications to a *BW25113 ΔldhA ΔackA* (BW25113 *ΔΔ*) background strain were applied in order to improve the conversion of formate into hydrogen. Sequential inactivation of the Fhl repressor hycA, and the uptake hydrogenases *hyaAB* and *hybBC*, were combined with overexpression of the Fhl activator fhlA. A first evaluation of strains took place in anaerobic serum bottles with ethanol, pyruvate and formate as main fermentation outputs. Due to the *ackA* knockout in *BW25113 ΔΔ*, NADH requirements for ethanol formation cannot be balanced by co-production of acetate, but are met by secretion of the intermediate metabolite pyruvate.

**Fig. 2:**
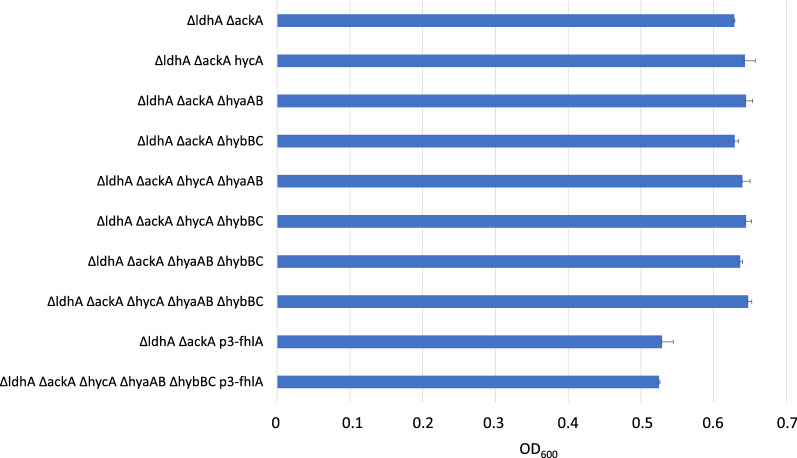
OD_600_ after 72 h of cultivation under anaerobic conditions with glucose as carbon source of a BW25113 *ΔldhA ΔackA* background strain containing additional KOs and/or overexpressing fhlA for improved hydrogen production. Initial OD600 was 0.2. Data and error bars indicate averages and standard deviations among duplicates

Neither the three individual knock-out events, nor a combination thereof, did have any effect on growth rates of the resulting strains when compared to their parental strain *BW25113 ΔΔ* (Fig. 2). After 72 h of cultivation, all strains reached an OD_600_ of around 0.64. Overexpression of fhlA was achieved by introduction of the *p3* promoter in front of the start codon of the native *fhlA*. This modification slightly affected growth of the double-knockout strain *BW25113 Δldh Δack p3-fhlA (BW25113 ΔΔ p3-fhlA*) as well as in the quintuple-knockout strain *BW25113 Δldh Δack ΔhycA ΔhybBC ΔhyaAB p3-fhlA* (*BW25113 ΔΔΔΔΔ p3-fhlA*) (Fig. 2). Overexpression of fhlA led to a reduced OD_600_ after 72 h, 15% lower compared to parental strains relying on native expression of fhlA.

**Fig. 3:**
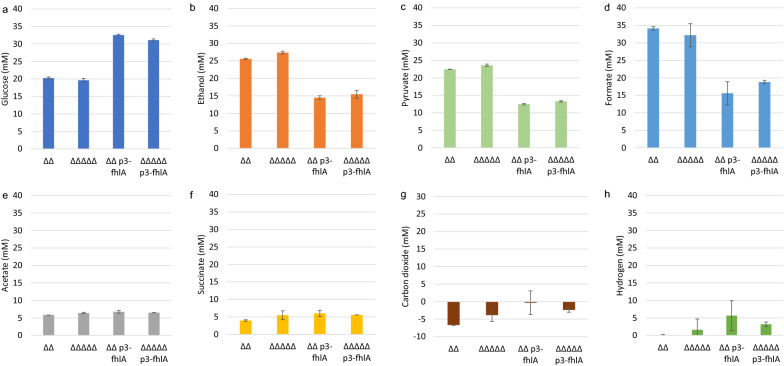
Concentrations of glucose and products after 72 h of anaerobic cultivation for strains with a *ΔldhA ΔackA (ΔΔ*) background and further modifications for improved hydrogen production, from left to right: inactivation of *hycA, hyaAB* and *hybBC* (*ΔΔΔΔ*), overexpression of *fhlA* (*ΔΔ p3-fhlA*) and a combination of knockouts and overexpression (*ΔΔΔΔ p3-fhlA*). For CO_2_ and H_2_, data represent calculated concentrations. Data show average values and standard deviations from biological duplicates

At the same time strains overexpressing fhlA consumed about 30% less glucose, resulting in less ethanol, pyruvate and formate production (Fig. 3a–d). Despite knocking out *ackA* some acetate production could not be avoided and reached levels around 6 mM for all strains tested (Fig. 3e). Succinate titers reached 3.96 ± 0.2 mM for the parental strain *BW25113 ΔΔ*, but were increased by 10% to 50% by strains with additional modifications towards hydrogen production, likely due to increased CO_2_ availability (Fig. 3f).

It is difficult to determine hydrogen and carbon dioxide gas production accurately in serum bottles. The effect of the genetic modifications on the production of both gasses was therefore estimated indirectly, by subtracting the amount of formate produced from the amount of ethanol plus acetate formed to obtain a calculated hydrogen concentration (mM). For estimating CO_2_, fixation for succinate synthesis was included, but not CO_2_ production associated with biosynthesis. This resulted in slightly negative calculated CO_2_ concentrations (Fig. 3g). While for the parental strain no H_2_ could be calculated, the other strains generated between 2 and 8 mM (Fig. 3h). However, variations in formate accumulation and conversion among duplicates led to large error bars in calculated concentrations.

**Fig. 4:**
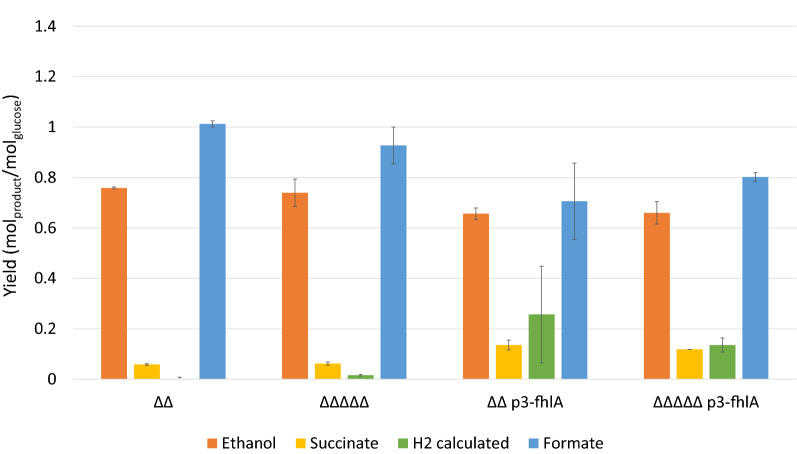
Product yield on glucose on selected products after 72 h of anaerobic fermentation for strains based on *ΔldhA ΔackA* (*ΔΔ*) with further modifications for improved hydrogen production, from left to right: inactivation of hycA, hyaAB and hybBC (*ΔΔΔΔΔ*), overexpression of fhlA (*ΔΔ p3-fhlA*) and a combination of knockouts and overexpression (*ΔΔΔΔΔ p3-fhlA*). Values are averages of two biological replicates and error bars represent standard deviations

Ethanol yields on glucose dropped by 12% for strains overexpressing fhlA in respect to *BW25113 ΔΔ* and *BW25113 ΔΔΔΔΔ* for which yields of about 0.8 mol_ethanol_/mol_glucose_ were obtained (Fig. 4). However, succinate yields significantly increased and doubled for *BW25113 ΔΔ p3-fhlA* and *BW25113 ΔΔΔΔΔ p3-fhlA* (*p* < 0.05). For strain *BW25113 ΔΔΔΔΔ* the hydrogen yield on glucose was only 0.02 mol_hydrogen_/mol_glucose_. Both strains overexpressing fhlA reached a higher yield, around 0.1 and 0.25 mol_hydrogen_/mol_glucose_, respectively. However, due to variations in the replicas only *BW25113 ΔΔΔΔΔ* and *BW25113 ΔΔΔΔΔ* p3-fhlA showed significant increase in hydrogen yields (*p* < 0.05).

Concluding, the effect of the subsequent inactivation steps in strain *BW25113 ΔΔΔΔΔ* remains elusive while overexpression of fhlA supports hydrogen production. On the other hand, overexpression causes a reduction in biomass formation and slower glucose consumption.

### Combining hydrogen gas and ethyl acetate production

After initial screening experiments and indirect performance assessments, three strains were generated with the purpose of co-producing hydrogen and ethyl acetate from glucose as carbon source. Strains *BW25113 Δldh Δack* p3-fhlA (*BW25113 ΔΔ p3-fhlA*), *BW25113 Δldh Δack ΔhycA ΔhyaAB ΔhybBC *(*BW25113 ΔΔΔΔΔ*) and *BW25113 Δldh Δack ΔhycA ΔhyaAB ΔhybBC p3-fhlA *(*BW25113 ΔΔΔΔΔ p3-fhl*) were equipped with the plasmid that encoded the ethanol acetyltransferase, pET26b:*T7/LacI-trEat1 Wan N13 *(trEat1) and gene expression was induced by 0.01 mM IPTG. Anaerobic ethyl acetate and hydrogen co-production were assessed in pH-controlled 1.5-L bioreactors with a continuous N_2_ gas flow of 100 mL/min coupled to online MS measurements of the off-gas. In this way stripped ethyl acetate, as well as produced CO_2_ and H_2_ could be measured and quantified.

**Fig. 5:**
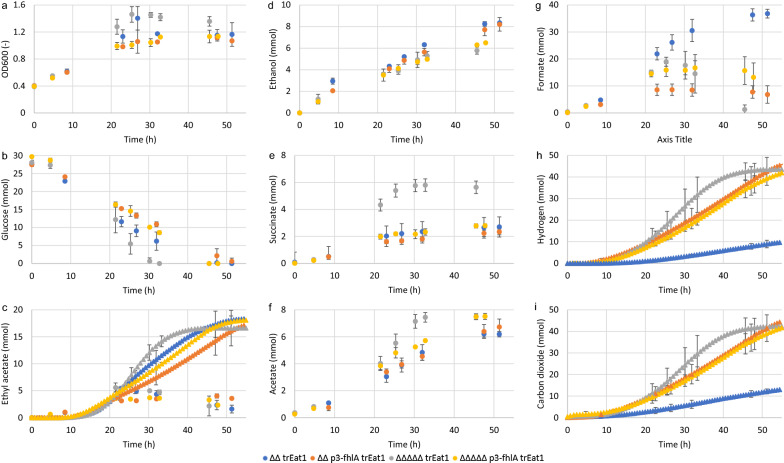
Fermentation profile of four strains engineered for ethyl acetate and hydrogen co-production in pH-controlled bioreactors with continuous gas stripping. Strains based on *ΔldhA ΔackA* (*ΔΔ*) with further modifications for improved hydrogen production, from left to right: inactivation of hycA, hyaAB and hybBC (*ΔΔΔΔΔ*), overexpression of fhlA (*ΔΔ p3-fhlA*) and a combination of knockouts and overexpression (*ΔΔΔΔΔ p3-fhlA*) producing trEat1 Wan N-13 were induced by 0.01 mM IPTG and cultivated under anaerobic conditions in minimal medium with 55 mM glucose as carbon source.. Experiments were performed as biological duplicates; error bars represent the standard deviation. Circles—compounds in liquid broth, triangle—compounds in off-gas

Similar to observations during the serum bottle experiments, overexpression of fhlA led to a decrease in maximum OD_600_ and slower glucose conversion (Fig. 5a, b). In contrast, however, knocking out the formate hydrogen lyase repressor and both uptake hydrogenases improved overall fermentation performance of *BW25113 ΔΔΔΔΔ* trEat1 including a reduced total fermentation time by about 35%. Expression of Eat1 and synthesis of ethyl acetate in a redox-balanced way, apparently lifted the earlier observed NADH shortage and therefore prevented pyruvate excretion almost completely (Additional file 2: Figure S1). Gas stripping kept overall ethyl acetate levels in the fermentation broth well below 10 mmol and resulted in a cumulative amount of stripped ethyl acetate near to 20 mmol (Fig. 5c). Formation of other by-products such as ethanol, acetate and succinate were mostly similar among all strains and did not exceed 10 mmol per compound (Fig. 5d–f). However, *BW25113 ΔΔΔΔΔ* trEat1 did accumulate more than twice as much succinate as the remaining strains. Formate secretion was reduced for all engineered strains, while H_2_ and CO_2_ accumulated to 4-times higher levels than the control strain without modifications in Fhl regulation or hydrogenases (*BW25113 ΔΔ* trEat1) (Fig. 5g–i).

**Fig. 6:**
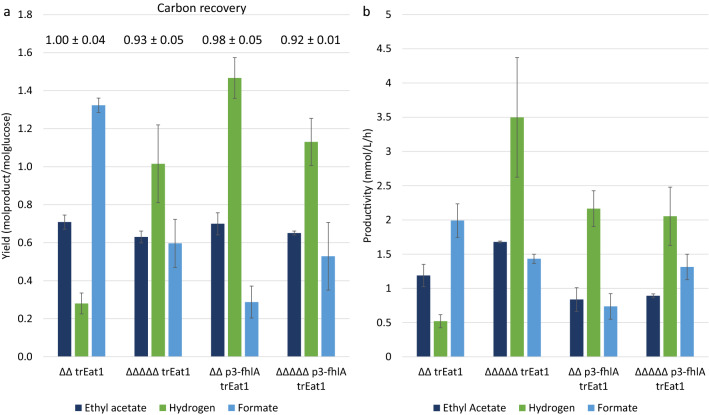
Effect of modifications towards improved hydrogen production on product yields and productivities for main fermentation products, with from left to right: inactivation of hycA, hyaAB and hybBC, overexpression of fhlA and a combination of knockouts and overexpression. Strains producing trEat1 *Wan* N-13 were induced by 0.01 mM IPTG and grown under anaerobic conditions in minimal medium containing 55 mM glucose using pH-controlled bioreactors with 0.5-L working volume. **a** Product yields for ethyl acetate, hydrogen and formate in mol_product_/mol_glucose_ after glucose depletion. The numbers above the bars represent the carbon recovery of the fermentations. **b** Volumetric productivities for ethyl acetate, hydrogen, and formate in mmol/L/h. Experiments were performed as biological duplicates or triplicates; error bars represent the standard deviation. *trEat1* truncated Eat1 Wan N-13

With respect to product yields, no significant differences in ethyl acetate yields on glucose could be found. With yields ranging from 0.63 ± 0.03 to 0.71 ± 0.04 mol_ethyl acetate_/mol_glucose_ about 70% of the pathway maximum was reached (Fig. 6a). The overall carbon yield Y_Carbon_ was 92% or higher for all strains (Additional file 1: Table S1). Knocking out *hycA, hyaAB, hybBC*, as well as overexpressing fhlA significantly improved hydrogen yields, reaching 50% and more of the pathway maximum. For the strain overexpressing fhlA (*BW25113 ΔΔ p3-fhlA* trEat1), the highest hydrogen yield was obtained with 1.47 ± 0.11 mol_hydrogen_/mol_glucose_, corresponding to 73% of the pathway maximum.

Despite that the product yield for ethyl acetate was rather similar, productivity of ethyl acetate did differ among the different strains. *BW25113 ΔΔΔΔΔ* trEat1 showed an improved ethyl acetate production by 41% (*p* = 0.052), while both fhlA overexpression strains showed a drop in productivity by 25–30%, which was however, not statistically significant (Fig. 6b). Regarding the co-production of hydrogen, all modifications led to a significant increase in conversion of formate into hydrogen and concomitantly CO_2_ (*p* < 0.05). The highest hydrogen productivity of 3.5 mmol/L/h was reached by *BW25113 ΔΔΔΔΔ* trEat1. Unexpectedly, overexpression of fhlA led to hydrogen production rates of only 2 mmol/L/h regardless whether only fhlA was overexpressed or additional knockouts were carried out.

## Discussion

The current study demonstrates how anaerobic ethyl acetate production can be coupled to efficient hydrogen co-production, thereby improving overall fermentation performance of the system. With an ethyl acetate yield on glucose close to 70% of the pathway yield *E. coli* can compete with natural producers, like *K. marxianus* [13] and performs close to earlier reported values using a truncated version of *W. anomalus* Eat1 [3].

Inactivation of the uptake hydrogenases (*hyaAB* and *hybBC*) and the Fhl repressor (*hycA*) led to 4-times higher hydrogen production rates relative to the control strain. While other studies found that those modifications did not negatively affect growth rates, here, the strain performance was even slightly improved during batch reactor fermentations [17]. This is likely a consequence of reduced formate concentrations, that may impose inhibitory effects to the cells [37].

Hydrogen yields realized by modified strains ranged from 1–1.47 mol_hydrogen_/mol_glucose_, thus the improvements are comparable to earlier reported values around 1.15–1.8 mol_hydrogen_/mol_glucose_ [6, 16, 19, 29, 36]. Overexpression of the Fhl activator fhlA using the *p3* promoter led to the highest hydrogen yields on glucose in *BW25113 ΔΔ p3-fhlA* trEat1, with a product yield of 1.47 mol_hydrogen_/mol_glucose_, respectively. However, for this strain also reduced biomass formation and reduced production rates of hydrogen and ethyl acetate were observed. In previous research, overexpression of fhl from a low copy number plasmid improved growth rates and hydrogen production from formate [35]. Also on glucose no impact of overexpression was noted using an IPTG-inducible expression system while the plasmid insertion itself did reduce the growth rate of the strain and also impacted growth rates during aerobic cultivation on formate [16, 17]. Therefore, fine-tuning the overexpression with different promoters or inducible expression systems, combined with adaptation seems necessary to keep the hydrogen overexpression strains competitive. While the applied modifications reportedly improve hydrogen (co-)production, there are still options to inactivate formate exporters (focA) or other formate-consuming enzymes including formate dehydrogenase-*N* (FdnG), dehydrogenase-O (FdoG), or nitrate reductase A (NarG) that positively impacted hydrogen production [16].

In the mentioned studies, efficient hydrogen-producing strains also carried an *frdAB* inactivation to eliminate succinate formation, which should be considered when optimizing further towards the maximum pathway yield of 2 mol_hydrogen_/mol_glucose_. Especially for strain *BW25113 ΔΔΔΔΔ* trEat1 the succinate yield was 2-times higher than the parental strain and may have masked the positive effects of hydrogen production as carbons were diverted from the intended co-product ethyl acetate.

Complete suppression of acetate formation is challenging and inactivation of *ackA* or *pta* often leads to a reduction in acetate accumulation only [11, 32]. Inactivation of the full *ackA-pta* operon, could help to lower acetate accumulation to negligible amounts [27, 30]. Additionally, acetate may originate from Eat1 thiolysis or esterase side-activities converting ethyl acetate or acetyl-CoA into acetate [3, 23]. Eliminating side-activities by protein engineering may be one way to overcome this drawback of Eat1. Here, we applied gas stripping to remove ethyl acetate more efficiently and reduce the residence time in the fermentation broth. Next to product degradation, product toxicity is another factor tackled with this strategy [8, 14]. Like most products, ethyl acetate can accumulate to toxic concentrations, thereby imposing inhibitory effects on the cells. For *E. coli* the threshold is estimated for ethyl acetate titers above 110 mM [34]. While this concentration was not and could not be reached under the tested conditions, gas stripping will become more important once the process is further upscaled. Moreover, the production of H_2_ and CO_2_ instead of formate, also benefits from gas stripping and enables continuous removal of both products of interest.

Low hydrogen yields during fermentation in expression hosts like *E. coli* combined with a mix of other fermentation products is a major drawback in microbial hydrogen production [18, 28]. Besides efficient production of hydrogen, production of only one other main fermentation product remains challenging Especially high-yield production of ethanol is often limited by NAD(P)H availability. Since NAD(P)H is only produced during the EMP pathway (GAP oxidation), ethanol formation can only amount to 1 mol_ethanol_/mol_glucose_, with the concomitant formation of 1 mol_acetate_/mol_glucose_. Higher ethanol yields requires additional NAD(P)H. Various engineering approaches have been used to generate extra NAD(P)H; Sundara Sekar et al. [29] employed a partial pentose phosphate pathway, which resulted in co-production of ethanol and hydrogen, with limited by-products formation or loss of growth, reaching yields for ethanol and hydrogen on glucose of 1.4 mol_ethanol_/mol_glucose_ and 1.88 mol_hydrogen_/mol_glucose_, respectively. Others made use of a pyruvate dehydrogenase instead of the pyruvate formate lyase yielding more NAD(P)H and reaching ethanol yields of 1.8 mol_ethanol_/mol_glucose_ [38]. The latter obviously occurs at the expense of formate or hydrogen. Thus, optimal co-production of hydrogen and one other product requires a redox-balanced acetyl-CoA conversion. The production of ethyl acetate as demonstrated here enables such redox neutral acetyl-CoA conversion and simultaneously co-production of hydrogen at its theoretical maximum of 2 mol_hydrogen_/mol_glucose_. With the co-production of ethyl acetate and hydrogen from glucose of 0.71 mol_ethyl acetate_/mol_glucose_ and 1.47 mol_hydrogen_/mol_glucose_ for strain *BW25113 Δldh Δack p3-fhlA* pET26b:Eat Wan N13, we successfully provide a first outlook on the applicability of this strategy towards another industrially relevant compound. Especially with respect to green hydrogen, co-production strategies offer an elegant way to improve the economic feasibility of a microbial production route and should be further pursued.

## Conclusion

Modification of the Fhl regulation system is an effective way to improve hydrogen production in *E. coli*. Overexpression of the Fhl activator *fhlA*, but also the inactivation of the Fhl repressor *hycA* and hydrogenases 1 and 2 by knocking out *hyaAB* and *hybBC* improved hydrogen production fourfold. During anaerobic fermentation of *BW25113 Δldh Δack p3-fhlA* pET26b:*T7/LacI-trEat1 Wan* N-13 on glucose 70% of the pathway yields for ethyl acetate and hydrogen, 0.695 mol_ethyl acetate_/mol_glucose_ and 1.44 mol_hydrogen_/mol_glucose_, respectively, were obtained. Cultivation of *BW25113 Δldh Δack ΔhycA ΔhyaAB ΔhybBC* pET26b: *T7/LacI-trEat1 Wan* N-13 led to highest ethyl acetate and hydrogen production rates, being 1.41- and 4-fold higher than the parental strain that mainly accumulated formate. Coupled to in situ product removal by gas stripping both products can efficiently be produced and recovered, offering attractive downstream processing opportunities for co-production of bio-based ethyl acetate and green hydrogen by *E. coli*.

## Methods

### Strain and plasmid construction

All strains and plasmids used can be found in Tables 1 and 2. Generation of genomic knockouts and insertion of *p3*-promoter [21] was achieved by CRISPR–Cas9 [5]. To generate the corresponding pTarget plasmid, a sequence containing gRNA module and the homologous sequences of 50 bp immediately upstream the start codon and downstream the stop codon were ordered as synthetic gBlocks (IDT) (Additional file 1: Table S2). For insertion of the p3-promoter sequence, the homologous sequences were located 35 bp upstream and beginning with the start codon for the downstream sequence. Using 2X HiFi assembly master mix (NEB) according to manufacturer’s instructions plasmids were assembled and propagated in competent NEB® 5-alpha cells. The pET26b:*T7/LacI-trEat1 Wan N-13* plasmid was inserted by following instructions from the Mix&Go *E. coli* Transformation Kit (ZYMO Research). PCR amplification was performed using Q5 polymerase (NEB).

**Table 1:** Strains used in this study

Strain	Characteristics	Source
*Escherichia coli* BW25113 (DE3)	Wild type with integrated DE3 lysogen	[32]
*Escherichia coli* BW25113 Δ*ackA*Δ*ldhA*	Disruption of lactate and acetate production (via *ackA*)	[9]
*Escherichia coli* BW25113 Δ*ackA*Δ*ldhA p3-fhlA*	Disruption of lactate and acetate production (via *ackA*) and overexpression of formate hydrogen lyase transcriptional activator (fhlA)	This study
*Escherichia coli* BW25113 Δ*ackA*Δ*ldhA*Δ*hycA*	Disruption of lactate and acetate production (via *ackA*) and inactivation of Fhl repressor (*hycA*)	This study
*Escherichia coli* BW25113 Δ*ackA*Δ*ldhA*Δ*hyaAB*	Disruption of lactate and acetate production (via *ackA*) and inactivation of uptake hydrogenase (hyaAB)	This study
*Escherichia coli* BW25113 Δ*ackA*Δ*ldhA*Δ*hybBC*	Disruption of lactate and acetate production (via *ackA*) and inactivation of uptake hydrogenase (hybBC)	This study
*Escherichia coli* BW25113 Δ*ackA*Δ*ldhA*Δ*hycA*Δ*hyaAB*	Disruption of lactate and acetate production (via *ackA*) and inactivation of Fhl repressor (hycA) and uptake hydrogenase (hyaAB)	This study
*Escherichia coli* BW25113 Δ*ackA*Δ*ldhA*Δ*hycA*Δ*hybBC*	Disruption of lactate and acetate production (via *ackA*) and inactivation of Fhl repressor (hycA) and uptake hydrogenase (hybBC)	This study
*Escherichia coli* BW25113 Δ*ackA*Δ*ldhA*Δ*hyaAB*Δ*hybBC*	Disruption of lactate and acetate production (via *ackA*) and inactivation of uptake hydrogenases (hyaAB, hybBC)	This study
*Escherichia coli* BW25113 Δ*ackA*Δ*ldhA*Δ*hycA*Δ*hyaAB*Δ*hybBC*	Disruption of lactate and acetate production (via *ackA*) and inactivation of Fhl repressor (hycA) and uptake hydrogenases (hyaAB and hybBC)	This study
*Escherichia coli* BW25113 Δ*ackA*Δ*ldhA*Δ*hycA*Δ*hyaAB*Δ*hybBC p3-fhlA*	Disruption of lactate and acetate production (via *ackA*) and inactivation of Fhl repressor (hycA) and uptake hydrogenases (hyaAB and hybBC) with overexpression of Fhl activator (fhlA)	This study
*Escherichia coli* T7 Express	fhuA2 [lon] ompT gal (λ DE3) [dcm] ∆hsdS λ DE3 = λ sBamHIo ∆EcoRI-B int::(LacI::PlacUV5::T7 gene1) i21 ∆nin5	NEB
*Escherichia coli* NEB® 5-alpha	*fhuA2 Δ(argF-lacZ)U169 phoA glnV44 Φ80 Δ(lacZ)M15 gyrA96 recA1 relA1 endA1 thi-1 hsdR17*	NEB

**Table 2:** Plasmids used in this study

Plasmid	Promoter	Gene/protein	Source
pET26b	LacI/*T7*	–	This study
pET26b:hWan trEat1 N-13	LacI/*T7*	Codon-harmonized *eat1* from *Wickerhamomyces anomalus* DSM 6766	[9]
pCas9	–		[5]
pTarget	–		[5]
pTarget-*hycA*	–		This study
pTarget-*hyaAB*	–		This study
pTarget-*hybBC*	–		This study
pTarget-p3	–		This study

### Cultivation

Strains were routinely cultured on LB medium with supplementation of spectinomycin (50 μg/mL) and/or kanamycin (50 μg/mL) when appropriate. Preculturing of strains was started by plating glycerol stocks stored at − 80 °C onto LB agar plates. From single colonies, overnight cultures for transformations or experiments were inoculated into 10 mL LB medium in a 50-mL tube and grown at 30 °C and 250 rpm. For pre-cultures and anaerobic experiments, 250-mL Erlenmeyer flasks or serum bottles were filled with 50 mL modified M9 medium consisting of M9 salts (Difco, 1X), glucose (55 mM), MgSO_4_ (2 mM), CaCl_2_ * 2 H_2_O (0.1 mM), MOPS (100 mM) and 1X trace elements and vitamin solutions based on [31]. The serum bottles were capped and flushed with nitrogen gas for anoxic conditions. From overnight cultures 1–2 mL were transferred to 50 mL modified M9 medium in 250-mL Erlenmeyer flasks and grown at 30 °C and 250 rpm. Strains for anaerobic experiments were inoculated as biological duplicates at an initial OD_600_ of 0.2 and incubated at 30 °C and 150 rpm [9].

### Batch reactor fermentation

Batch fermentations were performed in 1.5-L bioreactors (Applikon, The Netherlands) with a working volume of 0.5 L as described before [3]. Defined medium contained glucose (55 mM), (NH_4_)_2_SO_4_ (37.8 mM), KH_2_PO_4_ (22 mM), NaCl (171 mM), kanamycin (100 µg/mL), Na_2_SeO_3_ (0.3 mg/L) and 1X trace elements and vitamin solutions [31]. Eat1 gene induction was achieved by addition of 0.01 mM isopropyl β-d-1-thiogalactopyranoside (IPTG). Stirring at 400 rpm with a Rushton turbine was controlled by a ADI 1012 Motor Controller (Applikon), the target pH of 7 was maintained by automated addition of 3 M KOH solution and a temperature of 30 °C was achieved by a Thermo Circulator ADI 1018 (Applikon). Oxygen impermeable Marprene tubing (Watson-Marlow, UK) and a gas flow of 6 L/h N_2_ set the framework for anaerobic conditions. Pre-cultures were prepared as stated above and used to inoculate the reactors to a starting OD_600_ of 0.4. Liquid samples were taken regularly via a sampling port to assess optical density and composition of the fermentation broth. Metabolites were analyzed by high performance liquid chromatography (HPLC) and gas chromatography coupled to a flame ionization detector (GC). The off-gas composition was determined by online measurements of a δB Process Mass Spectrometer (MS, Thermo Scientific™, USA).

### Calculations

During anaerobic serum bottle experiments, H_2_ and CO_2_ production was estimated indirectly. Calculated H_2_ and CO_2_ concentrations (C in mol/L) were derived by assuming that significant production of either compound is solely attributed to Fhl activity, thus following the stoichiometric relation as shown in Eq. 1:

1 $$n {\mathrm{CH}}_{2}{O}_{2} \to n {\mathrm{CO}}_{2}+n {\mathrm{H}}_{2}$$

The deficit in formate measured and formate expected due to acetate and ethanol formation, combined with Eq. 1 leads to Eq. 2 with C_C_ (mol/L):

2 $${\mathrm{C}}_{{\mathrm{H}}_{2}}= \left({\mathrm{C}}_{{\mathrm{C}}_{2}{\mathrm{H}}_{5}\mathrm{OH}}+ {\mathrm{C}}_{{\mathrm{CH}}_{3}\mathrm{COOH}}\right)- {\mathrm{C}}_{{\mathrm{CH}}_{2}{\mathrm{O}}_{2}}$$

For CO_2_ calculations, also the incorporation of CO_2_ during the synthesis of succinate needs to be accounted for. Therefore, Eq. 2 is expanded to Eq. 3 for calculated CO_2_ concentrations (mol/L):

3 $${C}_{{\mathrm{CO}}_{2}}= \left({C}_{{\mathrm{C}}_{2}{\mathrm{H}}_{5}\mathrm{OH}}+ {C}_{{\mathrm{CH}}_{3}\mathrm{COOH}}\right)- {C}_{{\mathrm{CH}}_{2}{O}_{2}}- {C}_{{\mathrm{C}}_{4}{\mathrm{H}}_{6}{\mathrm{O}}_{4}}$$

In batch reactor, fermentations the off-gas composition was analyzed via online measurements via MS. Nitrogen, carbon dioxide, hydrogen, oxygen, ethanol and ethyl acetate fractions in the gas phase were considered and the cumulative amounts calculated as described in earlier research [3].

Carbon yields were estimated for all experiments according to Eq. 4 including glucose as substrate; ethyl acetate, ethanol, pyruvate, lactate, acetate, succinate, formate and CO_2_ as products and biomass based on a conversion factor of 0.3232 from OD_600_ to g/L dry weight [3] and assuming a biomass composition of CH_2_O_0.5_N_0.2_:

4 $${Y}_{\mathrm{Carbon}}=\frac{C-\mathrm{mol\,products\,formed}}{C-\mathrm{mol\,glucose\,consumed}}.$$

Volumetric productivities (*Q*_P_) were calculated in mmol/L/h by taking the slope of a linear trendline including at least four data points. For ethyl acetate productivity, only three data points could be included (Additional file 1: Table S2 and Additional file 3: Figure S2).

Statistical significance was assessed by using a two-sided Student’s *t*-test assuming equal variance and *p* < 0.05.

### Analytics

Liquid samples, including 50 mM propionic acid as internal standard, were analyzed with respect to glucose and organic acids using an Agilent 1290 LC II system (Agilent, USA), with an Agilent 1290 Infinity Binary Pump, Agilent 1290 Infinity Autosampler, Agilent 1290 Infinity diode array detector operated at 210 nm, and an Agilent 1260 Infinity RI detector operated at 45 °C [3]. The HPLC was operated with an Aminex HPX-97H (Bio-Rad, USA) column at 60 °C and 0.008 mM H2SO4 as mobile phase at 0.8 mL/min as flow rate.

Analysis of ethanol and ethyl acetate in the liquid phase was carried out by an Agilent 7890B gas chromatograph (Agilent, USA) equipped with a flame ionization detector (GC-FID) and an Agilent 7693 autosampler [9]. Samples were injected into a NukolTM column (30 m × 0.53 mm, 1.0 μm coating, Supelco, USA). Column temperature was maintained at 50 °C for 2 min, then increased to 200 °C at the rate of 50 °C/min, with a split ratio of 10. As internal standard 2 mM 1-butanol was added.

Online measurements of volatile compounds and gases removed from the reactor vessel by gas stripping were performed with an δB Process Mass Spectrometer (MS, Thermo Scientific™, USA) [3].

## Supplementary Information

**Additional file 1: Table S1.** Product and carbon yield in C-mol_product_/C-mol_glucose_ for strains cultivated in pH-controlled bioreactors with constant gas stripping after glucose depletion. Strains based on *ΔldhA ΔackA* (*ΔΔ*) with further modifications for improved hydrogen production, from left to right: inactivation of hycA, hyaAB and hybBC (*ΔΔΔΔΔ*), overexpression of fhlA (*ΔΔ p3-fhlA*) and a combination of knockouts and overexpression (*ΔΔΔΔΔ p3-fhlA*) producing trEat1 Wan N-13 were induced by 0.01 mM IPTG and cultivated under anaerobic conditions in minimal medium with 55 mM glucose as carbon source. **Table S2.** Product formation rates and R^2^ values of generated trendlines. **Table S3.** Information on gRNA and homologous sequences used for creating the pTarget vectors for genomic knockouts and insertions as described in Materials and Methods. gRNA – guide RNA, USR – upstream homologous region, DSR – downstream homologous region.

**Additional file 2: Figure S1.** Fermentation profile for pyruvate in pH-controlled bioreactors with continuous gas stripping. Strains based on *ΔldhA ΔackA* (*ΔΔ*) with further modifications for improved hydrogen production, from left to right: inactivation of hycA, hyaAB and hybBC (*ΔΔΔΔΔ*), overexpression of fhlA (*ΔΔ p3-fhlA*) and a combination of knockouts and overexpression (*ΔΔΔΔΔ p3-fhlA*) producing trEat1 Wan N-13 were induced by 0.01 mM IPTG and cultivated under anaerobic conditions in minimal medium with 55 mM glucose as carbon source. Experiments were performed as biological duplicates; error bars represent the standard deviation. Circles – compounds in liquid broth, triangle – compounds in off-gas.

**Additional file 3: Figure S2.** Product formation rates for strains co-producing ethyl acetate and hydrogen in pH-controlled reactors under anaerobic conditions. Rates are estimated by the slope of a linear trendline for cumulated product (mmol) per reactor volume (0.5 L) vs. time (h) to obtain rates in mmol/L/h. The rates and its corresponding R^2^ value per replicate are listed by compound in Additional file 1: Table S3.

## Data Availability

All data generated or analyzed during this study are included in this published article and its additional information files.

## Abbreviations

AAT: Alcohol acetyltransferase
AckA: Acetate kinase
*C*_C_: Concentration of compound C
DHAP: Dihydroxyacetone phosphate
Eat1: Ethanol acetyltransferase 1
EMP: Embden–Meyerhof–Parnas pathway
F6P: Fructose 6-phosphate
FBR: Fructose 1,6-bisphosphate
FocA: Formate transporter
FdoG: α-Subunit of formate dehydrogenase-*O*
FdnG: α-Subunit of formate dehydrogenase-*N*
Fhl: Formate hydrogen lyase
FhlA: Formate hydrogen lyase activator
G6P: Glucose 6-phosphate
GAP: Glyceraldehyde 3-phosphate
hyaAB: Subunits hyaA and hyaB of uptake hydrogenase 1
hybBC: Subunits hybB and hybC of uptake hydrogenase 2
hycA: Formate hydrogen lyase repressor
IPTG: Isopropyl β-d-1-thiogalactopyranoside
Kma: *Kluyveromyces marxianus*
Ldh: Lactate dehydrogenase
narG: α-Subunit of nitrate reductase A
pdc: Pyruvate decarboxylase
pfl: Pyruvate formate lyase
pta: Phosphate acetyltransferase
Q_P_: Volumetric productivity of product P (mmol/L/h)
trEat1: N-terminally truncated ethanol acetyltransferase 1
Wan: *Wickerhamomyces anomalus*
Y_Carbon_: Carbon yield (C-mol/C-mol)
