# Supplementary material for: Co-production of hydrogen and ethyl acetate in Escherichia coli
Source: Biotechnol Biofuels. 2021 Oct 1;14:192. doi: 10.1186/s13068-021-02036-3 (PMC8487115; doi:10.1186/s13068-021-02036-3)
Supplement: Supplementary file 1 — Additional file 1: Table S1. Product and carbon yield in C-molproduct/C-molglucose for strains cultivated in pH-controlled bioreactors with constant gas stripping after glucose depletion. Strains based on ΔldhA ΔackA (ΔΔ) with further modifications for improved hydrogen production, from left to right: inactivation of hycA, hyaAB and hybBC (ΔΔΔΔΔ), overexpression of fhlA (ΔΔ p3-fhlA) and a combination of knockouts and overexpression (ΔΔΔΔΔ p3-fhlA) producing trEat1 Wan N-13 were induced by 0.01 mM IPTG and cultivated under anaerobic conditions in minimal medium with 55 mM glucose as carbon source. Table S2. Product formation rates and R2 values of generated trendlines. Table S3. Information on gRNA and homologous sequences used for creating the pTarget vectors for genomic knockouts and insertions as described in Materials and Methods. gRNA – guide RNA, USR – upstream homologous region, DSR – downstream homologous region. [file 13068_2021_2036_MOESM1_ESM.docx]

Table S1: Product and carbon yield in C-mol_product_/C-mol_glucose_ for strains cultivated in pH-controlled bioreactors with constant gas stripping after glucose depletion. Strains based on *ΔldhA ΔackA* (*ΔΔ*) with further modifications for improved hydrogen production, from left to right: inactivation of hycA, hyaAB and hybBC (*ΔΔΔΔΔ*), overexpression of fhlA (*ΔΔ p3-fhlA*) and a combination of knockouts and overexpression (*ΔΔΔΔΔ p3-fhlA*) producing trEat1 Wan N-13 were induced by 0.01 mM IPTG and cultivated under anaerobic conditions in minimal medium with 55 mM glucose as carbon source.

|  | ΔΔ trEat1 | ΔΔΔΔΔ trEat1 | ΔΔ p3-fhlA trEat1 | ΔΔΔΔΔ p3-fhlA trEat1 |
| --- | --- | --- | --- | --- |
| Ethyl acetate | 0.47 ± 0.02 | 0.42 ± 0.02 | 0.47 ± 0.04 | 0.43 ± 0.01 |
| Ethanol | 0.10 ± 0.01 | 0.06 ± 0.01 | 0.10 ± 0.00 | 0.07 ± 0.00 |
| Pyruvate | 0.01 ± 0.01 | 0.00 ± 0.00 | 0.00 ± 0.00 | 0.00 ± 0.00 |
| Acetate | 0.08 ± 0.00 | 0.09 ± 0.00 | 0.08 ± 0.00 | 0.08 ± 0.00 |
| Lactate | 0.01 ± 0.00 | 0.00 ± 0.00 | 0.00 ± 0.00 | 0.00 ± 0.00 |
| Succinate | 0.05 ± 0.01 | 0.10 ± 0.01 | 0.04 ± 0.00 | 0.05 ± 0.00 |
| Formate | 0.22 ± 0.01 | 0.10 ± 0.02 | 0.05 ± 0.01 | 0.09 ± 0.03 |
| CO_2_ | 0.07 ± 0.01 | 0.16 ± 0.04 | 0.24 ± 0.02 | 0.19 ± 0.02 |
| Y_Carbon_ | 1.00 ± 0.04 | 0.93 ± 0.05 | 0.98 ± 0.05 | 0.92 ± 0.01 |

Table S2: Information on gRNA and homologous sequences used for creating the pTarget vectors for genomic knockouts and insertions as described in Materials and Methods. gRNA – guide RNA, USR – upstream homologous region, DSR – downstream homologous region

| pTarget | Part | Sequence |
| --- | --- | --- |
| hycA | gRNA | aagatggcgaagacaaacag |
|  | USR | gcacaaaaaatgcttaaagctggcatctctgttaaacgggtaacctgaca |
|  | DSR | gctgaggctttgcccgttttgcaggcgttacgcctgtttggggatgggcg |
| hyaAB | gRNA | tgaaattgtcaaaatccacg |
|  | USR | cataagcgcccggtgtcctgccggtgtcgcaaggaggagagacgtgcgat |
|  | DSR | cagcgaaggagaatcatcatgcaacagaaaagcgacaacgttgtcagcca |
| hybBC | gRNA | gccgcacattcagaacctgg |
|  | USR | gctggttcgtcgcaacaccaaaaacgaccatcacgacggaggagacgatc |
|  | DSR | atgcgtattttagtcttaggggtcggcaatattttgctgaccgatgaagc |
| P3-insertion | gRNA | tgaccttttgcaccgctttg |
|  | USR | gtatatgctaataaaattctaaatctcctatagttagtcaatgacctttt |
|  | insertion | agtctcaagcaccgctttgcggtgctttccaaaaaatttatttgcttattaatcatccggctcgtataatgtgtggaattcgaaagataaggaggtagcaca |
|  | DSR | atgtcatatacaccgatgagtgatctcggacaacaagggttgttcgacat |

Table S3: Product formation rates and R^2^ values of generated trendlines.

| Strain | | Ethyl acetate | | Formate | | Hydrogen | |
| --- | --- | --- | --- | --- | --- | --- | --- |
|  |  | Rate (mmol/L/h) | R^2^ | Rate (mmol/L/h) | R^2^ | Rate (mmol/L/h) | R^2^ |
| ΔΔ trEat1 | A | 1.3031 | 0.999 | 2.1632 | 0.9862 | 0.4527 | 0.9995 |
|  | B | 1.0721 | 0.9961 | 1.8179 | 0.9876 | 0.5875 | 0.9980 |
| ΔΔ p3-fhl trEat1 | A | 0.9592 | 0.9955 | 0.6044 | 0.9994 | 2.3493 | 0.9996 |
|  | B | 0.714 | 0.9996 | 0.8691 | 0.9983 | 1.9801 | 0.9988 |
| ΔΔΔΔΔ trEat1 | A | 1.6875 | 0.9658 | 1.385 | 0.9971 | 4.1158 | 0.9975 |
|  | B | 1.6687 | 0.9802 | 1.4792 | 0.9897 | 2.8801 | 0.9915 |
| ΔΔΔΔΔ p3-fhl trEat1 | A | 0.8703 | 0.9996 | 1.4444 | 0.9947 | 1.7516 | 0.9935 |
|  | B | 0.9112 | 0.9993 | 1.1814 | 0.9890 | 2.3546 | 0.9964 |
