# Supplementary figures and images for: Co-production of hydrogen and ethyl acetate in Escherichia coli
Source: Biotechnol Biofuels. 2021 Oct 1;14:192. doi: 10.1186/s13068-021-02036-3 (PMC8487115; doi:10.1186/s13068-021-02036-3)

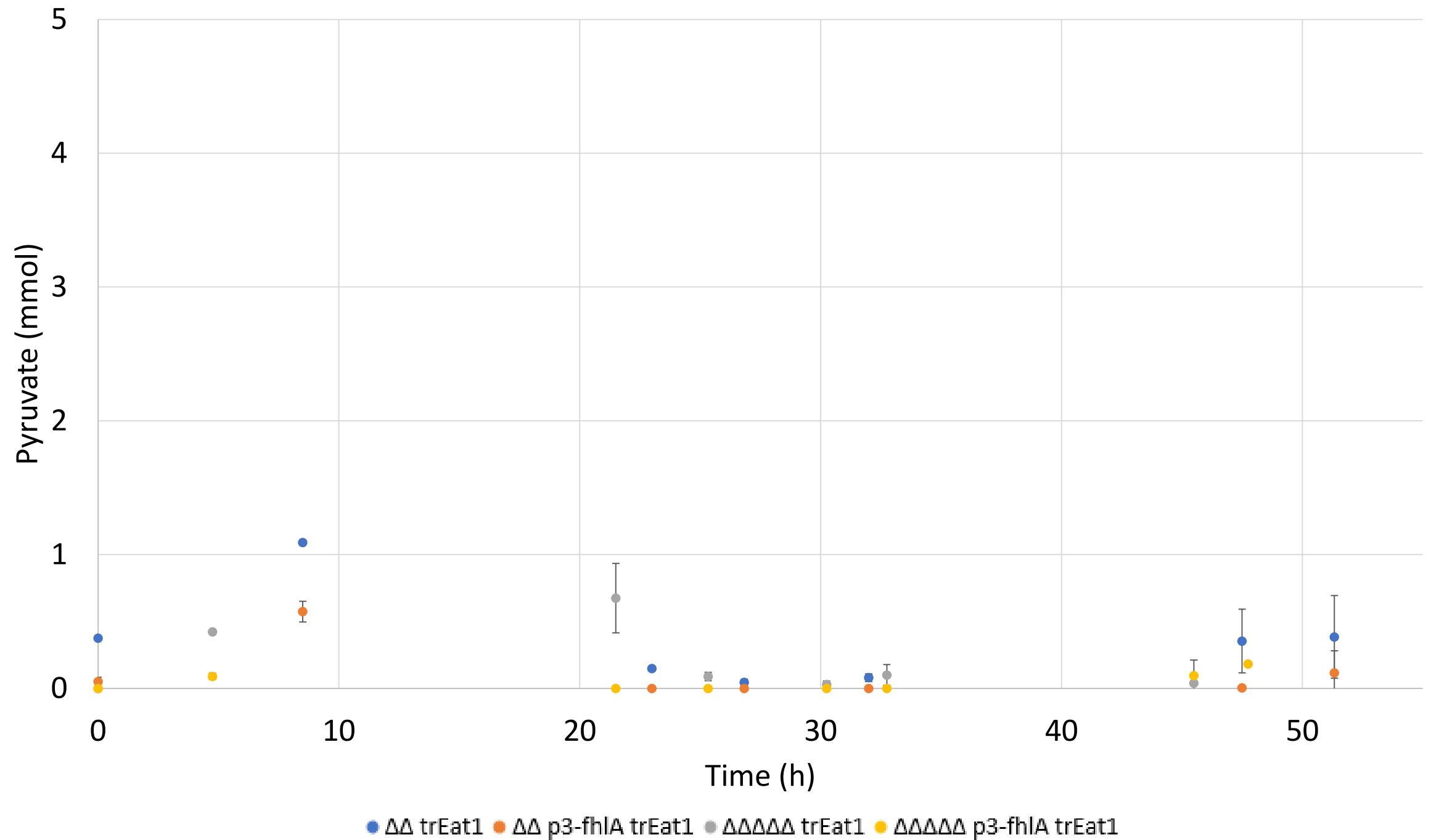

Supplement: Supplementary file 2 — Additional file 2: Figure S1. Fermentation profile for pyruvate in pH-controlled bioreactors with continuous gas stripping. Strains based on ΔldhA ΔackA (ΔΔ) with further modifications for improved hydrogen production, from left to right: inactivation of hycA, hyaAB and hybBC (ΔΔΔΔΔ), overexpression of fhlA (ΔΔ p3-fhlA) and a combination of knockouts and overexpression (ΔΔΔΔΔ p3-fhlA) producing trEat1 Wan N-13 were induced by 0.01 mM IPTG and cultivated under anaerobic conditions in minimal medium with 55 mM glucose as carbon source. Experiments were performed as biological duplicates; error bars represent the standard deviation. Circles – compounds in liquid broth, triangle – compounds in off-gas. [file 13068_2021_2036_MOESM2_ESM.pdf]
