# Supplementary material for: Co-production of hydrogen and ethyl acetate in Escherichia coli
Source: Biotechnol Biofuels. 2021 Oct 1;14:192. doi: 10.1186/s13068-021-02036-3 (PMC8487115; doi:10.1186/s13068-021-02036-3)
Supplement: Supplementary file 3 — Additional file 3: Figure S2. Product formation rates for strains co-producing ethyl acetate and hydrogen in pH-controlled reactors under anaerobic conditions. Rates are estimated by the slope of a linear trendline for cumulated product (mmol) per reactor volume (0.5 L) vs. time (h) to obtain rates in mmol/L/h. The rates and its corresponding R2 value per replicate are listed by compound in Additional file 1: Table S3. [file 13068_2021_2036_MOESM3_ESM.pdf]

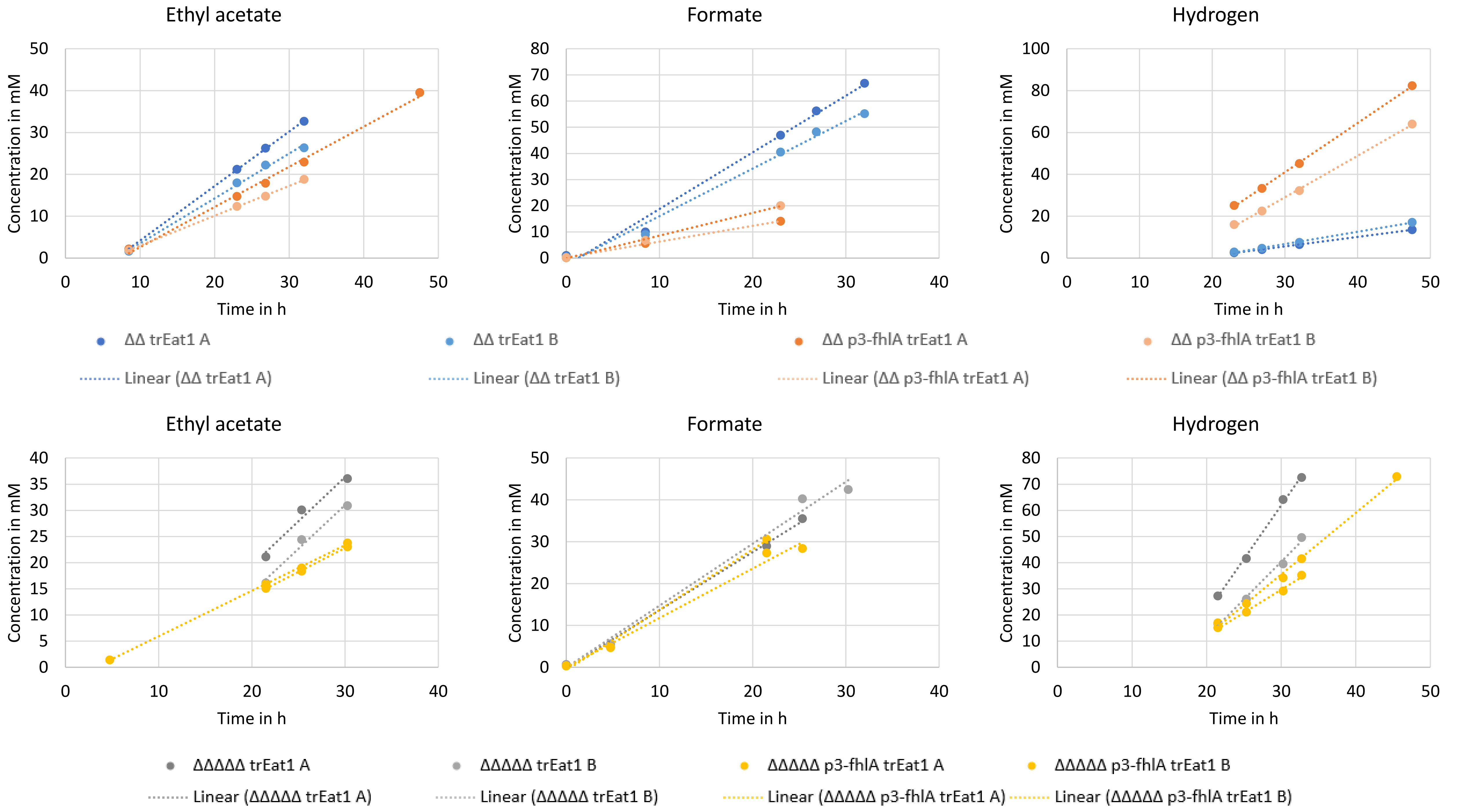
Ethyl acetate
Formate
Hydrogen
Concentration in mM
Time in h
ΔΔ trEat1 A
ΔΔ trEat1 B
ΔΔ p3-fhlA trEat1 A
ΔΔ p3-fhlA trEat1 B
Linear (ΔΔ trEat1 A)
Linear (ΔΔ trEat1 B)
Linear (ΔΔ p3-fhlA trEat1 A)
Linear (ΔΔ p3-fhlA trEat1 B)
Ethyl acetate
Formate
Hydrogen
ΔΔΔΔΔ trEat1 A
ΔΔΔΔΔ trEat1 B
ΔΔΔΔΔ p3-fhlA trEat1 A
ΔΔΔΔΔ p3-fhlA trEat1 B
Linear (ΔΔΔΔΔ trEat1 A)
Linear (ΔΔΔΔΔ trEat1 B)
Linear (ΔΔΔΔΔ p3-fhlA trEat1 A)
Linear (ΔΔΔΔΔ p3-fhlA trEat1 B)
